# It's cool to be stressed: body surface temperatures track sympathetic nervous system activation during acute stress

**DOI:** 10.1242/jeb.246552

**Published:** 2023-10-31

**Authors:** Paul Jerem, L. Michael Romero

**Affiliations:** ^1^ Groningen Institute for Evolutionary Life Sciences, University of Groningen, 9700 CC Groningen, The Netherlands; ^2^ Department of Biology, Tufts University, Medford, MA 02155, USA

**Keywords:** Body temperature, Non-invasive, Fight-or-flight, Sympathetic–adrenal–medullary system, Thermal imaging

## Abstract

The acute stress response can be considered the primary evolutionary adaptation to maximise fitness in the face of unpredictable environmental challenges. However, the difficulties of assessing physiology in natural environments mean that comparatively little is known about how response variation influences fitness in free-living animals. Currently, determining acute stress physiology typically involves blood sampling or cardiac monitoring. Both require trapping and handling, interrupting natural behaviour, and potentially biasing our understanding toward trappable species/individuals. Importantly, limits on repeated sampling also restrict response phenotype characterisation, vital for linking stress with fitness. Surface temperature dynamics resulting from peripheral vasomotor activity during acute stress are increasingly promoted as alternative physiological stress indicators, which can be measured non-invasively using infrared thermal imaging, overcoming many limitations of current methods. Nonetheless, which aspects of stress physiology they represent remains unclear, as the underlying mechanisms are unknown. To date, validations have primarily targeted the hypothalamic–pituitary–adrenal axis, when the sympathetic–adrenal–medullary (SAM) system is likely the primary driver of vasomotor activity during acute stress. To address this deficit, we compared eye and bill region surface temperatures (measured using thermal imaging) with SAM system activity (measured as heart rate variability via electrocardiogram telemetry) in wild-caught captive house sparrows (*Passer domesticus*) during capture and handling. We found that lower body surface temperatures were associated with increased sympathetic nervous system activation. Consequently, our data confirm that body surface temperatures can act as a proxy for sympathetic activation during acute stress, providing potentially transformative opportunities for linking the acute stress response with fitness in the wild.

## Teaser

**Summary:** Body surface temperature dynamics during acute stress tracked sympathetic nervous system activation. Consequently, sympathetic activity can be inferred non-invasively using infrared thermal imaging, overcoming many limitations of current measurement techniques.

## INTRODUCTION

The acute stress response equips individuals to deal with short-term challenges such as predation attempts and social aggression by rapidly triggering physiological processes that improve the probability of escape or competitive success (Romero and Wingfield, 2015). Consequently, the acute stress response can be considered as the primary evolutionary adaptation to maximise fitness in the face of unpredictable environmental challenges (Romero and Wingfield, 2015). But, despite such critical importance, we still know comparatively little about how variation in the acute stress response influences fitness in free-living animals, principally because of the difficulty of assessing acute stress in natural environments (Breuner et al., 2008).

The physiological acute stress response is complex and multi-faceted, but primarily involves sympathetic–adrenal–medullary (SAM) and hypothalamic–pituitary–adrenal (HPA) activation (Sapolsky, 2002). Techniques targeting point measurements of these systems typically involve blood sampling to assay hormone concentrations (Dantzer et al., 2014; Sheriff et al., 2011), or attachment of electrodes to assess cardiac activity (Casper, 2009; Gaidica and Dantzer, 2020; Hawkins, 2004). Integrated measurements of hormone metabolites have also been established (e.g. from faeces, urine, hair or feathers), although these usually only quantify HPA activity, and do not provide the temporal resolution necessary to study acute stress (Palme et al., 2005; Sheriff et al., 2011). Blood sampling in particular has provided extensive insight into patterns of physiological activity during acute stress, and how these relate to performance across a multitude of species (Romero and Wingfield, 2015). Nevertheless, both blood sampling and cardiac monitoring have limitations that restrict their usefulness in terms of linking acute stress with fitness. Firstly, they require individuals to be captured and handled. This can be perceived as a stressor, potentially altering subsequent physiology, behaviour and performance (Lynn et al., 2010; van Oers and Carere, 2007), and so confounding results. Secondly, confining investigations to trappable species and individuals potentially reduces generalisability and introduces bias (Garamszegi et al., 2009; Stuber et al., 2013). Thirdly, and possibly most importantly in this context, constraints on blood sampling frequency and often short datalogger battery lives impede efforts to track acute stress physiology over time [especially in smaller species, given their relatively low total blood volume (Fair et al., 2010) and load carrying capabilities (Casper, 2009)]. It is only through repeated measurements of acute stress that response phenotypes – the persistent traits on which selection acts – can be established, and fitness consequences determined (Romero and Gormally, 2019). Clearly then, there is a need for new methods that can overcome these issues.

One alternative sampling strategy is to instead examine traits that can be measured non-invasively, which link to acute stress physiology in a predictable way. In endotherms, body temperature is a particularly promising candidate trait for this purpose. At the onset of acute stress, the SAM system stimulates catecholamine hormone release within seconds, driving a rapid increase in heart rate (Carrive, 2006; Crestani, 2016; Sapolsky, 2002). At the same time, core body temperature rises (a phenomenon termed ‘stress-induced hyperthermia’; reviewed in Bouwknecht et al., 2007; Oka, 2018). Increased core body temperature during stress-induced hyperthermia is generated by a combination of increased metabolic heat output associated with higher heart rate (Nakamura, 2011), and reduced heat loss resulting from dorsomedial-hypothalamus-driven vasoconstriction at the body surface (Blessing, 2003; Nakamura, 2015; Oka, 2018; Oka et al., 2001). This latter feature of the acute stress response induces temperature reductions at the body surface that are of particular interest in the context of non-invasive sampling. Thermal imaging cameras can measure the temperature of object surfaces many times per second – without physical contact – by detecting the infrared radiation emitted by them (McCafferty, 2007, 2013; Tattersall, 2016). And, such cameras are becoming increasingly portable and affordable. As such, it may now be both possible and economical to infer acute stress physiology non-invasively in free-living animals from body surface temperature dynamics, overcoming many of the limitations of current methods.

There are encouraging signs that this is indeed the case, with increasing numbers of studies reporting characteristic body surface temperature changes during acute stress across a range of mammal (captive: Blenkuš et al., 2022; Faraji and Metz, 2020; Ludwig et al., 2007; MacRae et al., 2021; Riemer et al., 2016; Vianna and Carrive, 2005; Wongsaengchan et al., 2023; wild: Dezecache et al., 2017; Schraft and Clark, 2017) and bird species (captive: Edgar et al., 2011, 2013a,b; Giloh et al., 2012; Herborn et al., 2015, 2018; Knoch et al., 2022; Moe et al., 2017; Ouyang et al., 2021; Robertson et al., 2020a,b; Tabh et al., 2021; wild: Di Giovanni et al., 2022; Jakubas et al., 2022; Jerem et al., 2015, 2019). However, there are two critical issues with this body of work. First, the majority of these studies did not validate temperature data against other physiological measures of acute stress. And second, even where such steps were taken, the focus has been almost entirely on validation against the HPA axis (i.e. plasma glucocorticoid concentrations), when SAM system driven peripheral vasomotor activity is almost certainly the primary driver of surface temperature changes during acute stress.

To address this deficit, we compared surface temperatures of two body regions previously shown to exhibit temperature changes during acute stress in birds (see references above) – the ring of skin around the eye (*T*_eye_) and the bill (*T*_bill_) – with SAM system activity in wild-caught captive house sparrows (*Passer domesticus*) during the acute stress of capture and handling. We used heart rate variability, assessed via electrocardiogram (ECG) telemetry as our measure of sympathetic activation (Fischer and Romero, 2016). As reduced heart rate variability is indicative of SAM system activation (von Borell et al., 2007), we predicted a positive relationship between body surface temperature and heart rate variability during acute stress, such that lower heart rate variability (and therefore increased sympathetic activity) would be associated with lower body surface temperatures owing to associated increased peripheral vasoconstriction.

## MATERIALS AND METHODS

### Animals and housing

Seventeen wild adult house sparrows [*Passer domesticus* (Linnaeus 1758); nine females, eight males] were captured in Medford, MA, USA, between 23 April and 18 October 2022. Birds were housed in pairs and provided *ad libitum* food (Shafer Wild Bird Food, Shafer Seed, Jericho, NY, USA) and water. Individuals were allowed to acclimate to captivity for 38.6±3.3 days (mean±s.e.m, min.=19 days, max.=54 days) prior to ECG transmitter fitting (see ECG telemetry section below). Birds caught during October were checked to ensure post-breeding moult was complete before transmitter fitting. All birds were housed indoors, with experimental activities taking place in a controlled laboratory environment (mean±s.d. air temperature=20.8±0.3°C, min.=20.0°C, max.=21.3°C). All procedures were approved by Tufts Institutional Animal Care and Use Committee.

### Experimental design

Heart rate and heart rate variability (measured using ECG telemetry – see below), and eye and bill region surface temperatures (*T*_eye_ and *T*_bill_, respectively, measured using infrared thermal imaging – see below) were recorded simultaneously from each individual before, during and after the acute stress of capture and handling. Experimental runs (two birds each, tested simultaneously in separate cages in the same room) started at either 12:00 or 15:00 h, with a minimum of 48 h recovery for individuals undergoing multiple runs. Birds were moved from their home cage to a telemetry- and imaging-equipped experimental cage (30×24×20 cm width×height×depth, food and water supplied), had their ECG transmitters activated, and then were left undisturbed for 2 h prior to the start of testing to allow the physiological effects associated with being handled/moved to wash out. After 2 h (time=0 s), the experimenter entered the room, captured and held the bird within the cage (at 22.3±1.1 s), administered an injection (see below; at 70.5±2.7 s) and then released it from the hand (at 87.1±2.7 s). The experimenter then withdrew their hands from the cage, and closed the entrance, but remained standing by the cage, in view of the bird, until leaving the room at time=180 s. Continuous data recording started 60 s before the end of the wash-out interval, and continued until 30 min (1800 s) after stressor onset. Birds had their transmitters deactivated and were returned to their home cages immediately after recording had stopped at time=1800 s. A minimum sample size of 30 experimental runs was defined to match Jerem et al. (2019), which provided sufficient statistical power to detect the body surface temperature dynamics observed during acute stress in that study. The number of runs per individual was determined by ECG telemetry function and behaviour. Transmitter electrodes detached from muscle over time due to attachment fragility, combined with the free movement of the birds. Therefore, individuals were retired from the study once either electrode had detached and could not be repaired (no single electrode attachment was repaired more than once, and birds were allowed 48 h to recover from re-attachment before subsequent testing). Also, 19 runs where birds showed signs of distress (persistent escape or destructive behaviours) during the baseline period were abandoned, as measurements made during distress could not represent true baselines.

**Fig. 1.**
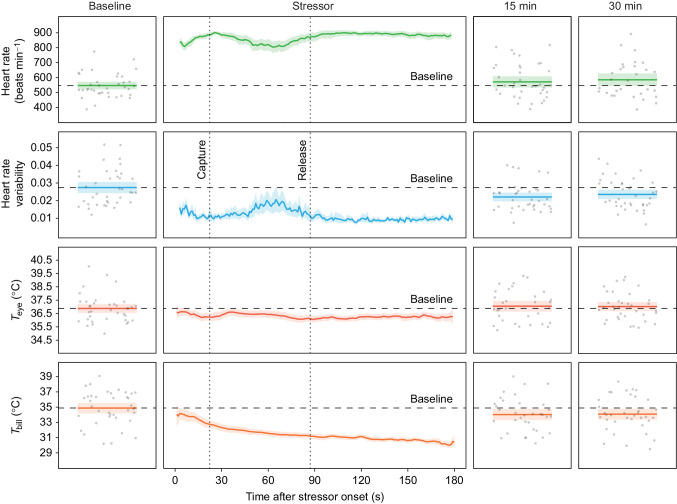
**Heart rate, heart rate variability and body surface temperatures recorded simultaneously from 17 captive house sparrows.** Sparrows were subjected to the acute stress of capture and handling over 38 experimental runs (mean±s.e.m. number of runs per bird=2.2±0.34, min.=1, max.=5). Grey circles are the mean (heart rate and heart rate variability) or maxima (maximum eye and bill region surface temperature, *T*_eye_ and *T*_bill_, respectively; see Materials and Methods) recorded during the 60 s before stressor onset (baseline), or 15 or 30 min after stressor onset for each run. Solid darker coloured lines and lighter surrounding shaded areas represent the mean±95% CI (bootstrapped). During the 3-min stressor period, data were interpolated to provide a mean value for each millisecond. See Materials and Methods for explanations of baseline/15 min/30 min period sample size variation.

Injections (saline control or pharmaceutical) were given as part of a parallel study not reported here. Statistical tests found no differences in heart rate variability, *T*_eye_ or *T*_bill_ between saline control and pharmaceutical treatments (see Table S1). Therefore, these data were assumed to be equivalent, and all were included in the plots and analyses. However, reduced heart rate was observed in Propranolol-treated birds (compared with controls and other treatments) during the 30 min period across eight tests. Consequently, these heart rate data points were removed from Fig. 1. All other heart rate data (where there were no statistical differences between saline control and pharmaceutical treatments) were assumed equivalent and included in Fig. 1.

### ECG telemetry

Cardiac activity monitoring followed the methods described by Fischer and Romero (2016) with minor modifications. Briefly, each bird was fitted with a ‘backpack’ mounted ECG telemetry transmitter (PhysioTel ETA-F10, Data Sciences International, St Paul, MN, USA). Backpacks consisted of an oval 3D-printed plastic base plate, with 5 mm wide polyester ribbons attached at each ‘corner’ to act as shoulder straps. The two upper straps were passed over the bird's shoulders, and the two lower straps under the wings, such that all met centrally on the chest, where they were sewn together to secure the backpack in place. The transmitter was sewn onto the plastic base within an elastane pouch, with its electrodes passing down to the bird through a central hole in the base plate. Looped electrode tips were passed subcutaneously through an incision made under the base plate hole, to incisions at the cervico-scapular junction and the caudo-dorsal region near the ilium, where the tips were twice sutured to muscle.

ECG and activity (a unitless measure of movement calculated from signal-to-noise-ratio) data were collected simultaneously from the transmitters during experimental runs using Dataquest ART v.4.0 software, controlling a PhysioTel RMC-1 receiver mounted onto the side of each experimental cage, connected via an SCI Data Exchange Matrix (Data Sciences International). ECG data were processed to extract heart rate and R wave to R wave intervals (RRI) using Ponemah P3 Plus (v4.80-SP4 Build 3260, Data Sciences International). P3 Plus automatically detects R waves in ECG data (representing depolarization of the main mass of the ventricles – the largest wave type in the ECG trace) and uses their positions in the time series to calculate the required parameters. As signal quality is variable and can interfere with R wave detection, all ECG traces were manually inspected, and R wave positions were corrected as necessary. Similarly, automated measurements of heart rate and RRI made across data gaps resulting from signal noise or poor electrode connection generated markedly atypical high or low readings when compared with surrounding data. Such errors were identified by plotting data against time, and erroneous data points were manually removed.

### Infrared thermal imaging

Body surface temperatures were measured throughout experimental runs using infrared thermal imaging cameras (Radiometric FLIR Boson 640 Professional, focal length=18 mm, thermal sensitivity <50 mK, frame rate=7.5 Hz, FLIR Systems, Wilsonville, OR, USA). Cameras were placed 70 cm from, and focused on, a dowel perch situated halfway between the cage front and rear, parallel to their sensor plane, such that birds within the experimental cage were always within the field of view and zone of focus. At this distance with the bird's head in profile, the eye region comprised approximately 130–180 pixels, and bill length (see below) was approximately 19–21 pixels (front and rear of cage, respectively). Individual frames were captured as 16-bit linear TIFFs using OpenCV (v4.5.5; https://opencv.org/) via Python (v3.8.13; www.python.org/). Regional body surface temperatures (*T*_eye_ and *T*_bill_) were manually extracted from TIFFs using FIJI ImageJ (v2.9.0; Schindelin et al., 2012). Maximum temperatures were used, as regional surface temperatures of small free-moving birds imaged against larger cooler areas are more likely to be underestimated than overestimated due to motion blur (for further details, see Jerem et al., 2015, 2019). *T*_eye_ was measured as the maximum temperature within an oval selection encompassing the ring of skin around the eye. *T*_bill_ was measured as the maximum temperature along a line drawn across the vertical centre of the bill, from directly under the nare to the anterior tip (Winder et al., 2020). To minimise error associated with head orientation (Tabh et al., 2021) and motion blur, temperatures were only extracted from sharply focused images where the bird's head was approximately in profile. Additionally, bill length was recorded for all body surface temperature measurements as a position index, to allow variation associated with camera–subject distance and remaining deviations in head position (from profile) to be accounted for (Winder et al., 2020). All temperature measurements were calibrated against an object of known temperature, and similar emissivity to the subject, placed within the camera's field of view (Jerem et al., 2019). An iButton Thermochron (DS1921G, Maxim Integrated, San Jose, CA, USA) set to record temperature once per minute was covered in insulation tape (Scotch Vinyl Electrical Tape, 3M, St Paul, MN, USA) and attached to the front of each experimental cage. ﻿The difference between the temperature logged by the iButton and the mean iButton surface temperature estimated by the thermal imaging camera was then used to correct *T*_eye_ and *T*_bill_. All iButtons were themselves calibrated against a NIST-certified calibrated temperature probe (Excursion-trac, Traceable Products, Webster, TX, USA) immediately prior to the start of the study.

### Data processing and statistical analyses

All further data processing and analyses were performed using R v4.3.0 (https://www.r-project.org/). Data analysed were from time periods defined relative to stressor onset: ‘baseline’ time=−60 to 0 s; ‘stressor’ time=0 to 180 s; ‘15 min’ time=840 to 900 s; ‘30 min’ time=1740 to 1800 s. We defined the stressor period as 180 s, as this appears to be the period exhibiting the most dynamic variation in surface temperatures during acute stress (Herborn et al., 2015; Jerem et al., 2015, 2019; Nord and Folkow, 2019). The baseline, 15 min and 30 min periods represented single points in time and so were reduced to a single value per individual per period. For heart rate and heart rate variability, this value was the mean of all measurements taken from an individual within a given period. For *T*_eye_ and *T*_bill_, the maximum was used, as this was expected to be the most accurate measurement made (for rationale, see Infrared thermal imaging section above). As the longer (180 s) stressor period was expected to be when most body surface temperature changes would be observed (from Jerem et al., 2019), these data were kept as multiple readings. During the stressor period, sample size was dictated by the number of body surface temperature measurements taken. All *T*_eye_ and *T*_bill_ measurements were matched with synchronous values for heart rate, heart rate variability, activity, position index and air temperature (from the iButton used in thermal image calibration – see above).

Heart rate variability was calculated as the coefficient of variation of RRI within a 2 s rolling window (window width visually identified as the best trade-off between smoothing and trace detail) using runner (package: runner v0.4.3; https://CRAN.R-project.org/package=runner). However, when the moving window passed over sharp increases in heart rate, heart rate variability also appeared to spike temporarily, as an artefact of the gradient between low and high heart rate variability falling within the window (and so resulting in a higher coefficient of variation). Consequently, any heart rate variability values higher than baseline in the first 10 s of the stressor period (where heart rate increased sharply due to stressor onset) were removed to avoid such artefacts. Incomplete transmitter data time series (due to signal noise and/or poor electrode connection) were linearly interpolated to give one value per millisecond using na.approx (package: zoo v1.8-23; Zeileis and Grothendieck, 2005).

*T*_eye_ and *T*_bill_ were filtered to further reduce negative error via a rolling maximum applied over a 5 s rolling window (window width visually identified as the best trade-off between smoothing and trace detail) using runner (package: runner v0.4.3). To ensure adequate representation of body surface temperature within each period, data for an individual in a given period were only included in the analyses when there were ≥5 measurements prior to filtering. This meant that within-period sample sizes were below the number of successful experimental runs (*n*=38) for eye region baseline (*n*=37), 15 min (*n*=32) and 30 min (*n*=35) periods, and for bill region 15 min (*n*=34) and 30 min (*n*= 36) periods, as bird posture/position prevented imaging the region of interest over sufficient frames in some runs.

The mean plot of all responses (Fig. 1) was created using ggplot (package: ggplot2 v3.4.2; Wickham, 2009) to draw individual panels, and patchwork (v1.1.2; https://CRAN.R-project.org/package=patchwork) to arrange them. Confidence intervals (95% CI) were bootstrapped with 10,000 resamples using boot (package: boot v1.3-28.1; https://CRAN.R-project.org/package=boot) for the baseline, 15 min and 30 min periods, and meanclboot (package: Hmisc v5.0-1; https://CRAN.R-project.org/package=Hmisc) within ggplot for the stressor period.

Relationships between *T*_eye_ or *T*_bill_ and heart rate variability were analysed using linear mixed-effects models specified with lme (package: lme4 v1.1-32; Bates et al., 2015). As well as the predicted association with heart rate variability, we expected body surface temperatures could vary with activity (through minor effects of motion blur, even on apparently sharp images or changes in convective heat exchange associate with movement), position index (Winder et al., 2020), air temperature (Aschoff, 1979) and sex (Clarke and Rothery, 2008). Therefore, full models were defined with either *T*_eye_ or *T*_bill_ as the response variable, and heart rate variability as the explanatory variable of interest, along with activity, position index, air temperature and sex as covariates. The full model random effect structure was the position of the experimental run within the sequence experienced by the individual, nested within individual, nested within experimental round, to account for order effects, repeated measures within individuals, and effects of minor cage design changes and/or differences in post-surgery recovery times between rounds. As temporal autocorrelation was diagnosed in the full model (using acf; Package ‘stats’), an exponential correlation structure was applied [selected as the structure generating the model with the lowest Akaike information criterion (AIC) when comparing equivalent full models each differing only in correlation structure between AR1, linear, Gaussian or exponential]. Subsequent model selection also followed an information-theoretic approach (Burnham and Anderson, 1998). Random structure selection between all possible combinations of random effects followed (Zuur et al., 2009), with a random structure of experimental run position within the sequence experienced by the individual, nested within individual providing the lowest AIC. Next, dredge (package: MuMIn v1.47.5; https://CRAN.R-project.org/package=MuMIn) was used to fit all possible models (with this random structure) for fixed-effect selection. Models within ≤6 AIC of the top performing model (excepting uninformative models within ≤2 AIC of the top model; Arnold, 2010) were then averaged using model.avg (package: MuMIn v1.47.5), with parameter 95% CIs calculated using confint (package: stats). Predictions and 95% CIs for marginal effects plots (ggplot; package: ggplot2 v3.4.2; Wickham, 2009) of the relationship between *T*_eye_ or *T*_bill_ and heart rate variability within averaged top models were calculated using modavgPred (package: AICcmodavg v2.3-2; https://CRAN.R-project.org/package=AICcmodavg). All other explanatories were set to their mean values. Model assumptions were checked by visually diagnosing residual distributions, and variance inflation factors <3 calculated *post hoc* for explanatory variables (vif; package: Car v3.1-2; Fox and Weisberg, 2019) suggested no issue with collinearity in the final models.

## RESULTS

Heart rate, heart rate variability and body surface temperatures (maximum eye and bill region: *T*_eye_ and *T*_bill_, respectively) were recorded from 17 individuals (nine females, eight males) over 38 experimental runs (mean±s.e.m. number of runs per bird=2.2±0.3, min.=1, max.=5, 20 runs with females, 18 runs with males). After filtering to retain only the most accurate values (see Materials and Methods), *T*_eye_ was measured 79.0±3.3 times (min.=34, max.=139) each run (baseline period: 15.7±1.1 times, min.=5, max.=32; stressor period: 36.4±2.1 times, min.=12, max.=68; 15 min period: 14.6±1.4 times, min.=3, max.=32; 30 min period: 15.2±1.5 times, min.=3, max.=38), whereas *T*_bill_ was measured from each individual 75.4±2.7 times (min.=38, max.=112) per run (baseline period: 16.3±1.4 times, min.=2, max.=40; stressor period: 29.6±1.9 times, min.=11, max.=54; 15 min period: 13.9±1.5 times, min.=1, max.=32; 30 min period: 17.1±1.4 times, min.=4, max.=35). Mean heart rate increased from 556.7±25.4 beats min^−1^ in the baseline period to around 900 beats min^−1^ within 15 s of the experimenters entering the aviary room (Fig. 1). Heart rate then remained between 800 and 900 beats min^−1^ for the rest of the stressor period (with heart rate lowering and recovering between these values during handling). After the stressor period, heart rate returned to baseline levels (indicated by CI overlap with the baseline period mean; Fig. 1) by the time of the 15 min (571.5±37.7 beats min^−1^) and 30 min periods (584.9±42.3 beats min^−1^). Mean heart rate variability exhibited an opposite pattern, dropping from 0.03±0.004 during the baseline period to approximately 0.01 within 15 s of stressor onset, and remaining there for the rest of the stressor period (also reflecting the pattern of heart rate change during handling). But, unlike heart rate, heart rate variability remained slightly below baseline during the 15 min (0.02±0.004) and 30 min periods (0.02±0.003). *T*_eye_ decreased to approximately 1°C below baseline (36.9±0.34°C) within 15 s of stressor onset, recovering to just below baseline by around 40 s, and subsequently declining again to stay around 1°C below baseline for the rest of the stressor period. Once stressor exposure had ceased, *T*_eye_ returned to baseline levels for the 15 min (37.1±0.4°C) and 30 min periods (37.0±0.33°C). In contrast, *T*_bill_ dropped consistently throughout the stressor period, ending around 4°C lower than baseline (34.9±0.7°C). *T*_bill_ also remained marginally below baseline during the 15 min (34.0±0.7°C) and 30 min periods (34.1±0.7°C).

**Fig. 2.**
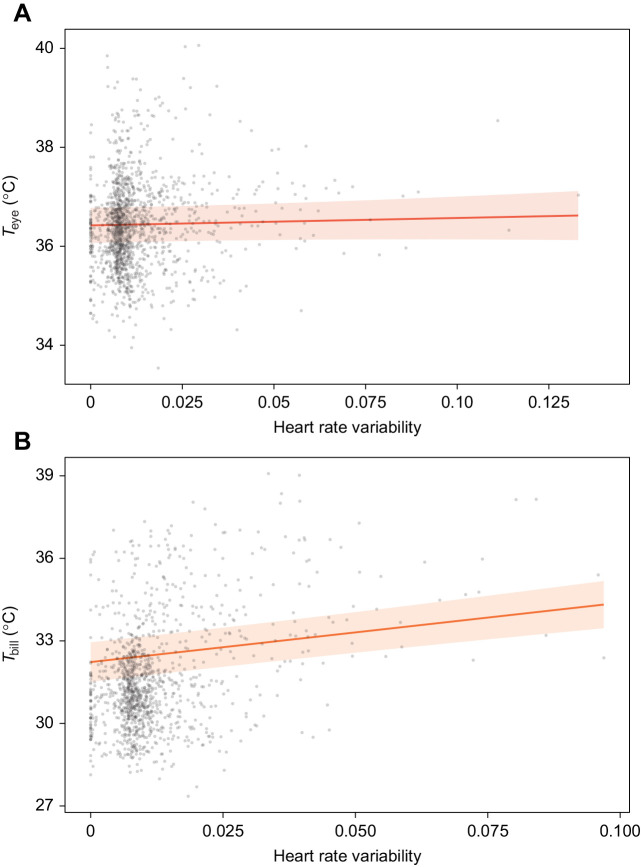
**Averaged top model predictions (see Tables 1 and 2) relating maximum body surface temperature with heart rate variability in 17 captive house sparrows.** Data are shown for maximum (A) eye and (B) bill region temperature immediately before and during acute stress (see Materials and Methods) over 38 experimental runs (mean±s.e.m. number of runs per bird=2.2±0.34, min.=1, max.=5). Grey circles are raw data points (eye region, *n*=1489; bill region, *n*=1231). Solid darker lines and lighter surrounding shaded areas represent model predictions±95% CI.

*T*_eye_ increased with heart rate variability and position index, and decreased with activity (Table 1). However, the association between *T*_eye_ and heart rate variability was weak (Fig. 2A), as it was only evident when coefficient averages were conditional on the predictor appearing in contributing top models (as opposed to more conservative full average calculations). As such, lower *T*_eye_ observed during the stressor period was predominantly driven by increased activity (with the association between activity and *T*_eye_ being approximately eight times stronger than that between *T*_eye_ and heart rate variability). *T*_bill_ exhibited a much more pronounced positive relationship with heart rate variability (Table 2, Fig. 2B), with the association between *T*_bill_ and heart rate variability being almost twice as strong as that between *T*_bill_ and activity. *T*_bill_ also increased with position index and decreased with activity and air temperature. Given that the strongest association between *T*_bill_ and any of its predictors was with heart rate variability, the primary driver of the ∼4°C decrease in *T*_bill_ over the stressor period was increased sympathetic nervous system activation. No differences in *T*_eye_ or *T*_bill_ were observed between the sexes.

**Table 1.**
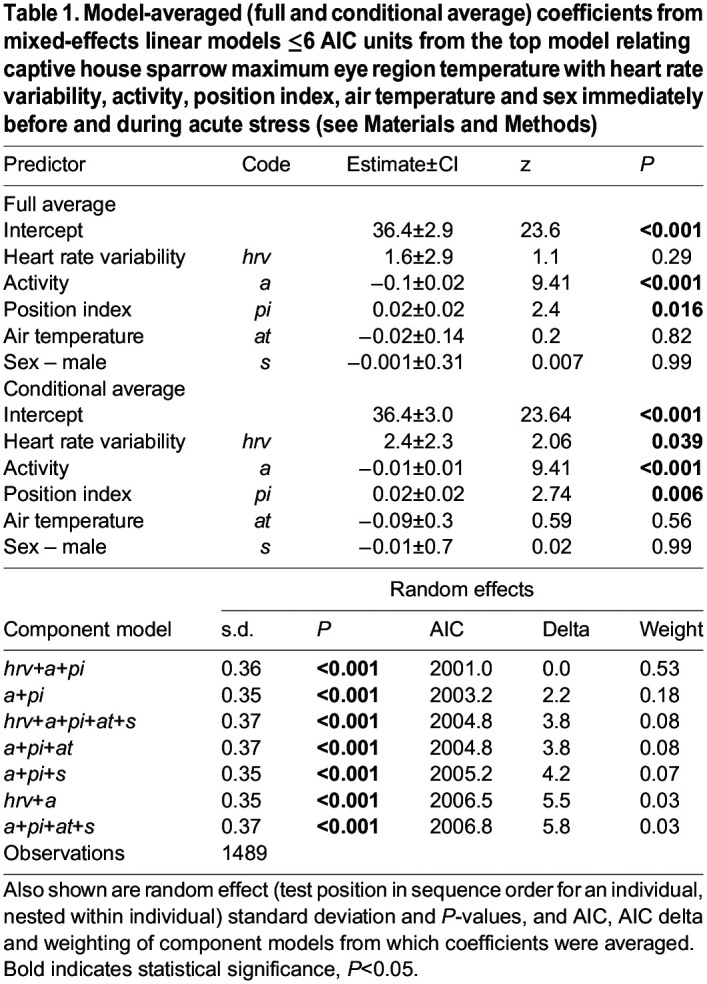
Model-averaged (full and conditional average) coefficients from mixed-effects linear models ≤6 AIC units from the top model relating captive house sparrow maximum eye region temperature with heart rate variability, activity, position index, air temperature and sex immediately before and during acute stress (see Materials and Methods)

**Table 2.**
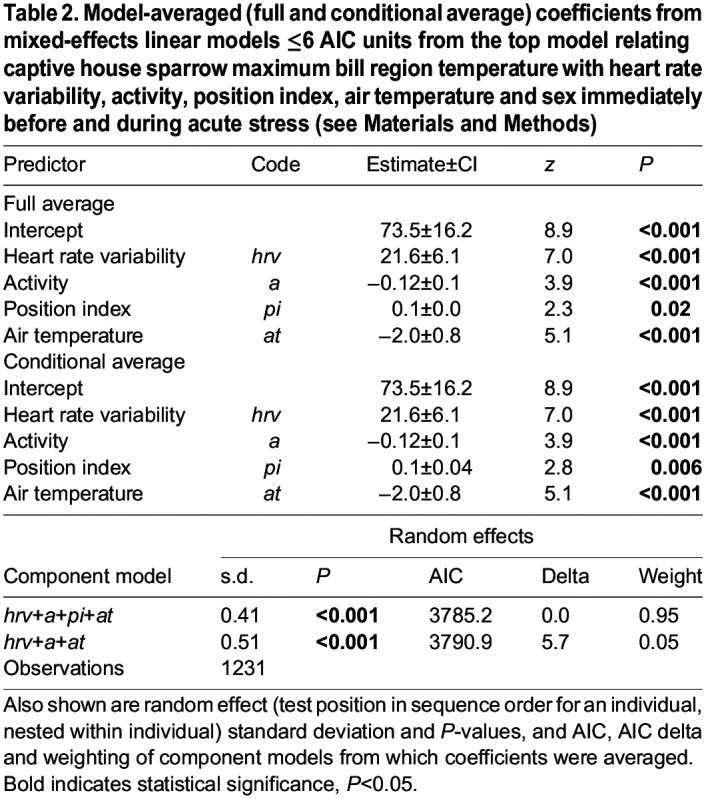
Model-averaged (full and conditional average) coefficients from mixed-effects linear models ≤6 AIC units from the top model relating captive house sparrow maximum bill region temperature with heart rate variability, activity, position index, air temperature and sex immediately before and during acute stress (see Materials and Methods)

## DISCUSSION

Body surface temperatures tracked sympathetic nervous system activation during acute stress, with lower body surface temperatures being associated with lower heart rate variability (and therefore higher SAM system activity). The effect of SAM system activity on bill surface temperature (*T*_bill_) was most pronounced, with a drop of approximately 4°C over the course of a 3-min stressor. *T*_bill_ also closely mirrored heart rate variability at 15 and 30 min after stressor onset. In contrast, the association with eye region surface temperature (*T*_eye_) was weaker, with only an approximate reduction of 1°C over the same period, driven predominantly by activity rather than SAM activation. Our analyses demonstrate that the relationship between *T*_bill_ and heart rate variability during our experiments was almost twice as strong as the relationship between *T*_bill_ and activity. As such, the *T*_bill_ changes we observed were primarily physiologically driven, and associated with sympathetic activity. Additionally, the tachycardia evident in Fig. 1 (top panel) must increase core temperature. Accordingly, under what were effectively constant ambient conditions (preventing temperature or wind-driven increases in peripheral heat loss), the simultaneous reductions in body surface temperatures we observed could only result from reduced heat transport from the body core to the surface. And, the only physiological process which could plausibly drive this effect is peripheral vasoconstriction. Consequently, our results validate body surface temperatures as a suitable target for inferring sympathetic activity during acute stress non-invasively using infrared thermal imaging in free-living endotherms. However, the substantial between-region differences in the strength of relationship between body surface temperatures and SAM activation we observed suggest that effort will need to be directed towards identifying the most effective group- and/or species-specific regions of interest for this purpose.

Few other attempts have been made to compare body surface temperatures with sympathetic nervous system activity during acute stress. Edgar et al. (2011, 2013b) reported decreased *T*_eye_ in response to puffs of air directed at the head of hens or their chicks, but without parallel reductions in heart rate variability. In both studies, heart rate changes between baseline and treatment periods were minimal, especially in comparison to those observed here. And, activity was not accounted for. Consequently, it remains possible that the reported *T*_eye_ reductions were driven at least partly by activity, given the effect of activity on *T*_eye_ we found, and that the air puffs did not appear to trigger sympathetic activation to any meaningful extent. Also, the air puff treatment itself may have contributed to falls in *T*_eye_ by increasing heat loss through convection, or evaporation from the eye, consistent with the greater decreases observed where air puffs were directed at the hens, rather than their chicks.

Although Vianna and Carrive (2005) did not examine heart rate variability, their simultaneous measurements of fear-conditioned rat paw and tail surface temperatures, heart rate and activity during acute stress exhibited remarkably similar patterns to those we report, at least until the end of stressor application. Both paw and tail surface temperatures dropped immediately on stressor onset, and remained below baseline during stressor exposure. These temperature reductions were greater in magnitude than those we observed (paw=−7.5°C, tail=−5.3°C), with surface temperatures eventually approaching air temperature. Such differences could result from interspecies differences in peripheral vasomotor capacity. Or, they may be evidence of a ‘floor’ effect (Bouwknecht et al., 2007) in our birds. As our house sparrows were wild caught, unlike the captive-bred rats, the chronic stress of captivity (Fischer et al., 2018) may have already lowered surface temperatures prior to any acute stress exposure, limiting the scope for further reductions. Unlike the paws, where surface temperature returned to baseline after the stressor ceased (as with *T*_eye_ in the present study), post-stressor tail surface temperatures were higher than baseline. This was associated with a simultaneous drop in core body temperature (which had increased consistently throughout stressor application), suggesting the rats were actively dumping heat retained through peripheral vasoconstriction during the stressor period, to avoid overheating. Presumably, higher air temperatures than those experienced in our aviaries [26–27°C in Vianna and Carrive (2005) versus 21°C in the present study] necessitated such active thermoregulation. Notably, the rats' eye region surface temperatures were also measured, but did not appear to change during acute stress. However, this appears more likely related to a small sample size (*n*=6 individuals) providing insufficient power to detect a weak effect, rather than a lack of a relationship (which was reported in another rat study with a larger sample size; Wongsaengchan et al., 2023).

The patterns of body surface temperature change under acute stress we observed are broadly similar to those seen elsewhere. For example, Jerem et al. (2015, 2019) reported immediate drops in *T*_eye_ followed by a recovery and subsequent decline during trapping and handling of wild blue tits (*Cyanistes caeruleus*), albeit much larger in magnitude than those observed among the house sparrows in this study. The contrasting scale of surface temperature changes is most likely related to air temperatures differing from those in our aviaries (7.7±0.6°C, min.=0.7° C, max.=11.2°C). Our comparatively small *T*_eye_ reductions in response to acute stress are also in agreement with the hypothesis that such changes may play a thermoregulatory role (Robertson et al., 2020a). The house sparrows in the present study were kept at air temperatures approximately 1.5–4°C below their lower critical temperature (the lower edge of their thermoneutral zone) (Anderson, 2006; Nzama et al., 2010). Given Robertson et al.'s (2020a) observation that reductions in stress-induced *T*_eye_ below the thermoneutral zone were minimised as the lower critical temperature was approached, smaller fluctuations in our study than those observed in overwintering wild birds would be expected. However, applying the same pattern exhibited by *T*_eye_ in Robertson et al.'s data to the much larger reductions we saw in *T*_bill_ would be less convincing. Doing so would mean house sparrow *T*_bill_ should drop by around 15°C at air temperatures only ∼7°C below those at which we made our measurements. This seems implausible, as it would result in negative temperature values for *T*_bill_. It follows then, that the temperature response of the bill in relation to the thermoneutral zone is likely to differ from that of the eye region. Nonetheless, the relationship between bill *T*_eye_ and *T*_bill_ responses does match those found by Tabh et al. (2021) and Moe et al. (2017) in that the *T*_bill_ response was also considerably more pronounced than for *T_eye_* in individuals subjected to capture and handling, below thermoneutrality. But, despite taking place at the similar air temperatures relative to lower critical temperatures as the birds in our study, the scope of surface temperature reductions in these studies was considerably smaller than those we report, possibly due to surface-to-volume ratio differences between the contrastingly sized species.

As our experiments took place below the thermoneutral zone, and we did not directly measure vasomotor activity, it remains possible that the surface temperature changes we report resulted from other processes (e.g. changes in convective heat loss related to movement), although this seems unlikely. It may be assumed that below the thermoneutral zone, vasoconstriction is immediately at its full extent, meaning further stress-related vasoconstriction would not be possible. However, the vasomotor response to cooling is gradual (Aoki et al., 2003; Owens et al., 2002) and complex, involving multiple processes such as narrowing of blood vessels and shunting of blood past capillary beds via arteriovenous anastomoses (Hales et al., 1978; Walløe, 2016). Additionally, the birds were only marginally below their thermoneutral zone. Therefore, thermogenesis alone could plausibly have compensated for ambient conditions so close to thermoneutral. Moreover, house sparrows are the most widely distributed species on the planet (Anderson, 2006). As such, they must be able to cope with the considerable variation in ambient temperatures found within their range. This also suggests that maximal vasoconstriction would be improbable at temperatures only slightly below thermoneutrality. Furthermore, any change in surface temperature associated with movement (whether from changes to convective heat exchange or motion blur) was accounted for statistically by our inclusion of activity in our analyses. Consequently, the relationship we report between heart rate variability and surface temperature cannot be a result of variation in convective heat exchange. Equally, the existence of surface temperature changes during stress resulting from vasomotor activity is long established (e.g. Solomon et al., 1964), with a growing body of research seeking to exploit this process to infer the physiological stress response (cited in the Introduction). Among this work are multiple studies where patterns of surface temperature change during acute stress are evident in animals restrained within controlled environments during measurements (e.g. Cabanac and Aizawa, 2000; Cabanac and Guillemette, 2001; Herborn et al., 2015; Ludwig et al., 2007; Moe et al., 2017; Nakayama et al., 2005; Tabh et al., 2021). Given minimal animal or air movement during these studies, changes in convective heat exchange cannot be responsible for the surface temperature dynamics reported.

The variance in the relationship between surface temperature and heart rate variability we report is admittedly relatively large. This is primarily because surface temperature changes during our experiment were cumulative over time, whereas changes in heart rate variability were not. Also, it is possible that during the 15 and 30 min periods, the combination of physiological processes contributing to surface temperatures differed from that during the stressor period, which may have confounded the relationship between surface temperarures and heart rate variability. As well as vasomotor activity at the periphery, acute stress induces core hyperthermia (Oka, 2018), which cannot be maintained without risking homeostatic overload/failure (Romero et al., 2009). As a result, where heat retention appears to be the purpose of the initial physiological response we observed, a later shift towards heat dissipation may be necessary to maintain homeostasis, via contrasting combinations of vasomotor activity and thermogenesis. Nevertheless, the fact that we were still able to detect a highly significant association (in the case of the bill) despite these factors is remarkable, and most likely due to the large numbers of repeated measures possible when using thermal imaging. Despite this, we do not advocate for the usefulness of body surface temperatures as an indicator of sympathetic activity at any single point in time. Instead, the fitness consequences of the acute stress response are much more likely to relate to patterns of sympathetic activation over time (e.g. how quickly an individual mounts a response, how long the response lasts, or the overall magnitude of the integrated response – i.e. the area under the temperature curve).

From the data we present, and that of a number of other studies (Dezecache et al., 2017; Edgar et al., 2013a; Faraji and Metz, 2020; Herborn et al., 2015; Schraft and Clark, 2017; Tabh et al., 2021; Vianna and Carrive, 2005; Wongsaengchan et al., 2023), it seems clear that different body regions will exhibit distinct relationships between their surface temperatures and sympathetic nervous system activation. Accordingly, identifying the most suitable target region(s) within a group or species will be a key preliminary task for studies wishing to employ body surface temperature as a proxy for acute stress. Exactly which attributes drive the strongest relationships between surface temperatures and sympathetic activity is currently unclear. Nonetheless, the most influential factors would be expected to comprise some combination of specific tissue function, vascular connectivity (Ruger Porter and Witmer, 2016; Verduzco-Mendoza et al., 2021), thermal conductivity (Duck, 1990) or relative risk of blood loss through injury (Blessing, 2003). For instance, it may be the case that despite plentiful vasculature and arteriovenous anastomoses (Ruger Porter and Witmer, 2016) – direct connections (shunts) between small arteries and veins which facilitate redirection of peripheral blood flow – the eye's proximity to the brain means the need to maintain brain temperature (Kiyatkin, 2019; Wang et al., 2016) limits the range of possible surface temperature change in that region. Conversely, the bill's non-thermoregulatory roles in foraging, preening, vocalisation and magnetoreception (Martin, 2017; Tattersall et al., 2016) appear unlikely to be appreciably negatively impacted by temporary restriction of blood flow to the bill surface. Therefore, redirection of blood away from the bill surface may incur lower costs than restricting blood flow to the eye region, and so provide the most economical region of the two from which to divert resources or reduce heat loss during acute stress.

As with changes in HPA function, and many other measures of neuroendocrine activity such as plasma concentrations of oxytocin, vasopressin, prolactin and opioid peptides (Paul et al., 2005), it remains possible that sympathetically driven fluctuations in surface temperature may primarily reflect arousal. As such, the ability of infrared thermal imaging to distinguish highly arousing, but differently valenced states (e.g. acute stress versus sexual attraction, or anticipation of a valuable reward) may be limited. Indeed, some studies have suggested this may be the case, demonstrating little or no differentiation in temperature responses between valences (Ermatinger et al., 2019; Kano et al., 2016; Moe et al., 2012). Nevertheless, valence may be revealed by contrasting regional patterns of temperature change (Dezecache et al., 2017; Ioannou et al., 2015; Kuraoka and Nakamura, 2011), and incorporation of behavioural and other contextual information (usually collected incidentally when recording thermal video) would be expected to allow valence to be inferred. Consequently, this constraint is unlikely to present a major problem when using body surface temperatures to infer SAM system activation during acute stress.

In terms of associations between body surface temperatures and non-sympathetic covariates, the *T*_eye_/*T*_bill_ position index relationships we report confirm the utility of bill length as a straightforward method for parsing out ‘technical’ variance linked with head angle and camera–subject distance (Winder et al., 2020). Equally, the negative association between activity and *T*_eye_/*T*_bill_ appears to usefully reveal – and account for – negative effects of motion on temperatures measured from even apparently sharp thermal images (a biological cause for this relationship seems unrealistic given the heat generated during exercise). The lack of a positive relationship between *T*_eye_/*T*_bill_ and air temperature in our data is somewhat surprising. But, minimal variance in air temperature during the tests (mean±s.d.=20.8±0.3°C, min.=20.0°C, max.=21.3°C) are likely to have rendered the usually observed association impossible to detect (at least with our sample sizes). This hypothesis is supported by analogous findings in studies employing similar techniques in lab settings (Ouyang et al., 2021; Wongsaengchan et al., 2023). That we did not detect sex differences in the relationship between sympathetic activation and *T*_eye_/*T*_bill_ may relate to our relatively small sample sizes (male=9, female=8), and the fact that we only analysed the general relationship rather than response patterns. Studies reporting sex differences in body surface temperatures during acute stress have tended to be those using more animals and/or examining specific response features (Blenkuš et al., 2022; Faraji and Metz, 2020; Knoch et al., 2022; Robertson et al., 2020a; Wongsaengchan et al., 2023; although see Jakubas et al., 2022; MacRae et al., 2021; Ouyang et al., 2021).

In conclusion, our data confirm that body surface temperatures can act as a proxy for sympathetic nervous system activation during acute stress. This proxy can be measured non-invasively using infrared thermal imaging, overcoming many limitations of current assessment techniques. As such, thermal imaging of body surface temperatures has transformative potential for improving our understanding of how wild animals use stress to adapt to changing environments. Without health, welfare or technological restrictions on repeated measures, thermal imaging provides clear opportunities to track response development through time, establish detailed individual response phenotypes, and link acute stress physiology with fitness. Given that stress diverts resources towards survival and away from reproduction (Boonstra et al., 1998), it may even be possible to apply this individual-derived information in ‘ecological surveillance’, modelling community-level changes to identify at-risk populations before declines in numbers occur. The highly conserved nature of SAM system anatomy among endotherms suggests the method will be broadly applicable once regulatory differences are accounted for (Romero and Gormally, 2019). But, it will be necessary to confirm exactly how the full range of possible ambient temperatures (especially those above the lower critical temperature) might affect the relationship between body surface temperatures and sympathetic activity before geographically widespread implementation. Probably the biggest current limitation lies in needing to keep animals within the camera's field of view and zone of focus during situations that will commonly involve target individuals fleeing from the scene. This situation will undoubtedly improve as more capable cameras become available over time. Until then, targeting the most suitable study systems is likely to yield the most effective data collection. Particularly amenable situations might involve larger animals, which are easier to track on camera (especially drone mounted), breeding birds, which are likely to remain on the nest during acute stress (alongside offering convenient access to fitness data), cryptic species that freeze rather than flee, and lekking species that compete for sexual partners within relatively confined areas. Perhaps less limiting, but still somewhat burdensome, is the labour still currently required to extract body surface temperatures from many thousands of images. Plainly, developing open-source machine learning data extraction tools which do not require prohibitively large training datasets would be highly beneficial in this sense.

## Supplementary Material

10.1242/jexbio.246552_sup1 Supplementary information
