# Supplementary material for: It's cool to be stressed: body surface temperatures track sympathetic nervous system activation during acute stress
Source: J Exp Biol. 2023 Oct 31;226(20):jeb246552. doi: 10.1242/jeb.246552 (PMC10629684; doi:10.1242/jeb.246552)
Supplement: Supplementary information [file jexbio-226-246552-s1.pdf]

Table S1. Coefficient estimates (± 95% confidence intervals), t-statistics and p-values from linear models comparing mean period (15 min or 30 min) heart rate or heart rate variability, or maximum eye or bill region surface temperature (*Teye* or *Tbill*, respectively) between individuals receiving a control saline injection and those experiencing one of four pharmaceutical treatments across 38 experimental runs.

Response	Period	Explanatory	Estimate±95%CI	t	p
Heart Rate	15 min	Treatment:Propranolol	-27.74±126.00	0.43	0.67
		Treatment:Phentolamine	49.80±117.87	0.83	0.67
		Treatment:ACTH	96.86±117.87	1.61	0.11
		Treatment:RU486	6.24±127.00	0.10	0.92
	30 min	Treatment:Propranolol	-128.88±89.37	2.83	**<0.01**
		Treatment:Phentolamine	-44.11±89.38	0.97	0.33
		Treatment:ACTH	64.48±92.51	1.37	0.17
		Treatment:RU486	-54.49±101.9	1.05	0.29
Heart Rate Variability	15 min	Treatment:Propranolol	0.01±0.02	1.53	0.103
		Treatment:Phentolamine	0.01±0.02	1.63	0.126
		Treatment:ACTH	0.01±0.01	1.26	0.209
		Treatment:RU486	0.00±0.01	0.19	0.853
	30 min	Treatment:Propranolol	0.00±0.02	0.24	0.81
		Treatment:Phentolamine	-0.01±0.02	0.74	0.46
		Treatment:ACTH	0.00±0.02	0.15	0.88
		Treatment:RU486	0.00±0.02	0.05	0.96
T_{eye}	15 min	Treatment:Propranolol	-0.28±1.3	0.42	0.67
		Treatment:Phentolamine	-0.42±1.25	0.66	0.51
		Treatment:ACTH	-1.09±1.21	1.77	0.08
		Treatment:RU486	-0.23±1.47	0.3	0.76
	30 min	Treatment:Propranolol	-0.18±0.71	0.34	0.74
		Treatment:Phentolamine	-0.30±30.77	0.55	0.58
		Treatment:ACTH	0.92±1.07	1.69	0.09
		Treatment:RU486	-0.13±1.30	0.2	0.85
T_{bill}	15 min	Treatment:Propranolol	-1.53±2.60	1.15	0.25
		Treatment:Phentolamine	0.09±2.43	0.08	0.94
		Treatment:ACTH	-0.61±2.42	0.49	0.62
		Treatment:RU486	0.21±2.59	0.16	0.87
	30 min	Treatment:Propranolol	-0.99±2.27	0.86	0.39
		Treatment:Phentolamine	0.14±2.27	0.12	0.91
		Treatment:ACTH	-0.68±2.34	0.57	0.57
		Treatment:RU486	-1.01±2.58	0.77	0.44

Journal of Experimental Biology • Supplementary information
